# CLRM-06. COMPARISON OF INDIVIDUALIZED ANTI-CANCER THERAPY REGIMENS RECOMMENDED BY A MULTIDISCIPLINARY MOLECULARLY-DRIVEN TUMOR BOARD IN A PEDIATRIC DIPG CLINICAL TRIAL (PNOC003) VERSUS THOSE SELECTED BY THE CNS-TAP TOOL

**DOI:** 10.1093/noajnl/vdab112.005

**Published:** 2021-09-21

**Authors:** Holly Roberts, Karthik Ravi, Allison Schepers, Bernard Marini, Cassie Kline, Sabine Mueller, Carl Koschmann, Andrea Franson

**Affiliations:** 1 University of Michigan Medical School, Ann Arbor, MI, USA; 2 University of Michigan Department of Clinical Pharmacy and Pharmacy Services, Ann Arbor, MI, USA; 3 Children's Hospital of Philadelphia, University of Pennsylvania Perelman School of Medicine, Philadelphia, PA, USA; 4 University of California San Francisco School of Medicine, San Francisco, CA, USA

## Abstract

Genetic sequencing of diffuse intrinsic pontine gliomas (DIPG) has revealed genomic heterogeneity, fueling an interest in individualized targeted therapies. A feasibility study, PNOC003: Molecular Profiling for Individualized Treatment Plan for DIPG (NCT02274987), was completed within the Pacific Pediatric Neuro-Oncology Consortium in which a multidisciplinary tumor board reviewed molecular and genomic profiling of each participant’s tumor to make targeted therapy recommendations. Separately, our team developed the Central Nervous System Targeted Agent Prediction (CNS-TAP) tool, which combines pre-clinical, clinical, and CNS penetration data with patient-specific genomic information to derive numeric scores for anticancer agents to objectively evaluate these therapies for use in patients with CNS tumors. We hypothesized that agents highly-scored by CNS-TAP would overlap with agents recommended by the PNOC003 tumor board. For each study participant, we retrospectively utilized the genomic profiling report to identify actionable alterations and incorporated these data into CNS-TAP to find the highest-scoring agents. We compared these CNS-TAP-recommended agents with recommendations from the tumor board for each of the 28 PNOC003 participants. Overall, 93% of patients (26/28) had at least one agent recommended by both the tumor board and CNS-TAP. Additionally, 38% of all agents (36/95) chosen by the tumor board were also selected by CNS-TAP. When only molecularly targeted anticancer agents were included in a sub-analysis, 60% of agents (34/57) were recommended by both methods. At present, we are prospectively evaluating the CNS-TAP tool within PNOC008: A Pilot Trial Testing the Clinical Benefit of Using Molecular Profiling to Determine an Individualized Treatment Plan in Children and Young Adults with High-Grade Glioma (NCT03739372). The CNS-TAP tool recommendations are shared during the PNOC008 molecular tumor board meetings once a consensus treatment recommendation has been reached. Subsequent analyses will focus on any adjustments in therapy decisions within the tumor board that result from the CNS-TAP tool output.

